# Association Between E-Health Literacy and OGTT Status at Study Enrollment Among Pregnant Women

**DOI:** 10.3390/healthcare14183026

**Published:** 2026-09-15

**Authors:** Tuğçe Taşar Yıldırım, Çiğdem Akçabay, Sevler Yıldız, Handenur Gökçe, Müberra Gökhan Çimenoğlu, Nevzat Gözel

**Affiliations:** 1 Department of Internal Medicine, Fethi Sekin City Hospital, Elazığ 23280, Türkiye; 2 Department of Obstetrics and Gynecology, Faculty of Medicine, Fırat University, Elazığ 23200, Türkiye; cagcabay@hotmail.com; 3 Department of Psychiatry, University of Health Sciences, Fethi Sekin City Hospital, Elazığ 23280, Türkiyehanndegokce@gmail.com (H.G.); 4 Department of Psychiatry, Elazığ Mental Health and Diseases Hospital, Elazığ 23200, Türkiye; muberragokhan@gmail.com; 5 Department of Internal Medicine, Faculty of Medicine, Fırat University, Elazığ 23200, Türkiye; drngozel@hotmail.com

**Keywords:** gestational diabetes mellitus, oral glucose tolerance test, screening, e-health literacy, pregnancy, health behavior

## Abstract

**Aim**: Gestational diabetes mellitus (GDM) is defined as glucose intolerance diagnosed during pregnancy and can lead to significant health problems for both the mother and the baby. We aimed to compare e-health literacy levels according to oral glucose tolerance test (OGTT) status at study enrollment and to examine the association between e-health literacy and OGTT status. **Materials and Methods**: The study included 45 pregnant women classified as OGTT-tested and 122 classified as OGTT-untested based on their OGTT status at study enrollment. All participants completed a sociodemographic data form and the e-health Literacy Scale (eHEALS). **Results**: A total of 167 pregnant women were included in the study; 45 (26.9%) had undergone an OGTT during their current pregnancy, while 122 (73.1%) had not. The mean total score on the eHEALS was 24.71 ± 8.58. No significant difference in eHEALS scores was observed between the OGTT-tested and OGTT-untested groups (25.04 ± 8.20 vs. 24.58 ± 8.75; Hodges–Lehmann median difference = 0.00, 95% CI: −3.00 to 4.00; *p* = 0.788; r = 0.027). In contrast, the proportion of women who had undergone an OGTT in a previous pregnancy was higher among those who underwent an OGTT in their current pregnancy (*p* < 0.001). eHEALS scores showed significant differences by age group (*p* = 0.019), education level (*p* < 0.001), and trimester (*p* = 0.014); the highest scores were observed in the ≥35 age group and among university graduates. eHEALS scores showed a positive correlation with age (rho = 0.240; *p* = 0.002) and a negative correlation with gestational week (rho = −0.225; *p* = 0.004). In the multiple linear regression model, having a college degree (B = 4.079; 95% CI: 0.244–7.915; *p* = 0.037) and trusting health information on the internet (B = 3.466; 95% CI: 0.421–6.511; *p* = 0.026) were independently associated with higher e-health literacy scores. Among the 45 pregnant women who underwent OGTT, 14 (31.1%) were diagnosed with GDM according to the International Association of Diabetes and Pregnancy Study Groups (IADPSG) criteria. In an exploratory subgroup analysis restricted to participants who underwent OGTT, eHEALS scores were higher among women diagnosed with GDM than among those without GDM (*p* = 0.042). **Conclusions**: In conclusion, in this sample, the level of e-health literacy did not differ significantly according to OGTT status at study enrollment.

## 1. Introduction

GDM is a metabolic disorder characterized by hyperglycemia diagnosed during pregnancy in women who did not have a diagnosis of diabetes prior to pregnancy, and it can affect maternal, fetal, and neonatal outcomes [1]. Recent estimates indicate that GDM affects approximately 14% of pregnancies worldwide, with its prevalence increasing in parallel with the global rise in obesity and type 2 diabetes. It is known that hyperglycemia associated with GDM is linked to outcomes such as hypertensive disorders of pregnancy, fetal macrosomia, birth trauma, and neonatal hypoglycemia [2,3,4]. Timely identification of GDM is important to enable appropriate glycemic management during pregnancy and to identify women and offspring who may benefit from long-term cardiometabolic risk assessment and follow-up [5].

The diagnosis of GDM is largely based on glucose screening and diagnostic tests performed during pregnancy [6]. Although the approaches and diagnostic strategies used for GDM screening may vary across guidelines, the OGTT remains one of the primary methods for evaluating glucose intolerance during pregnancy [6,7]. Conducting early screening tests, ensuring that pregnant women are properly informed, and performing a detailed evaluation for GDM are important components of antenatal care [8]. We know that the clinical benefit of screening tests depends not only on the test’s diagnostic performance but also on the target population’s participation in screening [9]. Pregnant women’s decisions to undergo GDM screening may be influenced by numerous factors, such as their level of GDM-related knowledge and health literacy, sociodemographic characteristics, and access to health care [10,11]. In the digital age, health information obtained from digital platforms must also be considered among these factors [12]. With advances in technology, the internet and related platforms have become major sources of health-related information during pregnancy; today, digital transformation has also become an integral part of pregnant women’s clinical decision-making processes.

E-health literacy encompasses the skills needed to access, evaluate, and appropriately use health information obtained from digital sources [13,14]. These competencies may be particularly important during pregnancy, when health-related decisions affect the well-being of both the mother and the fetus [15]. While e-health literacy may be associated with how pregnant women perceive and evaluate information regarding the potential benefits and risks of the OGTT, the relationship between e-health literacy and OGTT status has not yet been clearly established.

The reliability of online health information is important in the context of GDM screening. Women have the opportunity to access a wide range of information online regarding the necessity, safety, and effects of the OGTT; however, this information may at times be inconsistent with scientific evidence. Beyond access to health information, the ability to critically evaluate the reliability and appropriateness of the information obtained is also important [16,17]. High e-health literacy can facilitate the identification of reliable sources and a more accurate assessment of evidence-based health recommendations. In a cross-sectional study in Turkey involving 364 pregnant women in weeks 24–28, 55.7% of the participants agreed to undergo GDM screening; scores for health care, disease prevention, health promotion, and general health literacy were found to be higher in the screening group [10]. In contrast, another Turkish cross-sectional study found no association between the OGTT preference of 120 pregnant women and their general health literacy or level of perinatal anxiety; it was reported that the decision was most influenced by healthcare personnel [18].

Health-related decision-making processes are not merely the result of the level of knowledge acquired but are also influenced by psychosocial variables such as social support and the individual’s relationship with the healthcare system [19]. It is believed that e-health literacy among pregnant women diagnosed with GDM may be associated not only with educational level and health information-seeking behavior but also with psychosocial factors such as pregnancy-related anxiety and perceived social support [20,21]. Consequently, factors associated with OGTT results are not limited to obstetric and metabolic factors but may also include cognitive and psychosocial characteristics.

Previous studies have reported associations between health literacy and prenatal screening-related outcomes [22]. A recent study that assessed health literacy and attitudes toward prenatal screening among pregnant women reported that women who underwent screening tests had higher levels of health literacy and that pregnant women used not only healthcare professionals but also the internet and social media as important sources of information regarding prenatal screening [23]. However, evidence on the association between e-health literacy and OGTT status during pregnancy remains limited.

Accordingly, the aim of our study was to compare e-health literacy levels according to OGTT status at study enrollment and to evaluate the association between e-health literacy and OGTT status. It was hypothesized that e-health literacy levels would differ between the OGTT-tested and OGTT-untested groups. Additionally, the associations of sociodemographic and obstetric characteristics with e-health literacy and OGTT status at study enrollment were investigated.

## 2. Materials and Methods

Ethical approval for the study was obtained from the Firat University Non-Interventional Research Ethics Committee (Session No: 2026/10-07; Date: 9 July 2026). Written informed consent was obtained from all participants. This cross-sectional analytical study was conducted to evaluate the association between OGTT status at study enrollment and e-health literacy among pregnant women who presented to the Department of Obstetrics and Gynecology at Firat University School of Medicine. Data collection was conducted in July 2026, following ethics committee approval. A consecutive sampling approach was used. Eligible pregnant women attending the outpatient clinic during the study period were consecutively recruited after providing written informed consent. Pregnant women aged ≥ 18 years who were in their second or third trimester were included in the study; those under 18 years of age, those in their first trimester, or those who did not provide informed consent were excluded from the study. A total of 167 pregnant women were included in the study. Participants were categorized as OGTT-tested or OGTT-untested based on their OGTT status at the time of study enrollment. The 45 pregnant women who had undergone an OGTT during their current pregnancies were classified as the “OGTT-tested group,” while the 122 pregnant women who had not undergone an OGTT at the time of study enrollment were classified as the “OGTT-untested group.’’ As some women in the OGTT-untested group may not yet have reached the routine GDM screening window, OGTT-untested status was not considered equivalent to refusal of GDM screening. The reasons for not undergoing an OGTT at the time of study enrollment (refusal, not recommended, or other clinical reasons) were not systematically recorded.

The participants’ sociodemographic, obstetric, and clinical characteristics were recorded using a data form prepared by the researchers. The variables assessed included age, education level, place of residence, perceived economic status, employment status, number of previous pregnancies, gestational age, and trimester. Participants were also asked whether they used the internet to obtain health information and whether they trusted the health information they encountered online.

### 2.1. Assessment of E-Health Literacy

Participants’ e-health literacy levels were assessed using the Turkish version of the eHEALS, originally developed by Norman and Skinner and validated in Turkish by Uskun et al. in 2022 [24]. The eHEALS consists of items that assess an individual’s self-perceived competence in finding, evaluating, and using online health information. Each item is scored on a five-point Likert-type scale, with total scores ranging from 8 to 40; higher scores are interpreted as indicating a higher level of perceived e-health literacy [13,24]. The eHEALS was administered at the time of study enrollment. In the OGTT-tested group, eHEALS was therefore administered after the participants had undergone OGTT and, among women diagnosed with GDM, after the diagnosis had been established.

### 2.2. Evaluation of OGTT Status and GDM Diagnosis

Participants were divided into two groups according to their OGTT status at study enrollment: OGTT-tested and OGTT-untested. Among women in the OGTT-tested group, a 75 g OGTT was performed. GDM was diagnosed according to the criteria of the International Association of Diabetes and Pregnancy Study Groups (IADPSG) [25]. Accordingly, the diagnostic cutoff values were set at ≥92 mg/dL for fasting plasma glucose, ≥180 mg/dL for 1 h plasma glucose, and ≥153 mg/dL for 2 h plasma glucose. A result of at least one of these three measurements being equal to or above the relevant cutoff value was considered sufficient for a diagnosis of GDM.

The eHEALS was administered to all participants, and the scale scores were compared between the two groups. OGTT status was determined based on information regarding whether participants had undergone the test during their current pregnancy.

### 2.3. Statistical Analysis

Analyses were performed using IBM SPSS Statistics 26.0 (IBM Corp., Armonk, NY, USA). The normality of continuous variables was assessed using the Shapiro–Wilk test, skewness and kurtosis coefficients, and histograms. Normally distributed continuous variables were summarized as mean ± standard deviation; others as median (minimum–maximum); and categorical variables as frequency and percentage. The internal consistency of the eHEALS in the present sample was assessed using Cronbach’s alpha coefficient.

For the eHEALS comparison between the two groups, which was tested with the Mann–Whitney U test, the location shift between groups was expressed as the Hodges–Lehmann median difference, with its 95% confidence interval obtained by bootstrapping (2000 resamples), so that the reported estimate, confidence interval, hypothesis test, and effect size are methodologically consistent.

To compare two independent groups, an independent samples *t*-test was used for age; for gravida, gestational week, and eHEALS scores, the Mann–Whitney U test was used. The Kruskal–Wallis H test was used to compare more than two groups; when significant differences were detected, pairwise comparisons were performed using the Bonferroni correction. Categorical variables were compared using Pearson’s chi-square test, the chi-square test with Yates’ continuity correction, or Fisher’s exact test. The relationships among continuous variables were examined using Spearman’s correlation coefficient. Effect sizes were calculated using Cohen’s d or the rank-biserial correlation coefficient (r) for comparisons between two groups, ε^2^ for more than two groups, and Cramér’s V for categorical variables; 95% confidence intervals for d, r, and ε^2^ were obtained using the bootstrap method with 2000 repetitions.

In addition, a binary logistic regression analysis was performed with OGTT status at study enrollment as the dependent variable (1 = OGTT-tested, 0 = OGTT-untested). The eHEALS score was entered as the primary independent variable of interest, and age, education level, gestational week, previous-pregnancy OGTT, and trust in online health information were included a priori as clinically relevant covariates, in order to estimate the eHEALS–OGTT association adjusted for potential sociodemographic and obstetric confounders, rather than being selected on the basis of their univariate association with the outcome. Education level was modeled with two dummy variables (reference: primary education or below), previous-pregnancy OGTT with “no” as the reference, and trust in online health information with “no” as the reference. Results were reported as odds ratios (OR) with 95% confidence intervals. Model fit was evaluated with the omnibus likelihood-ratio test and the Hosmer–Lemeshow goodness-of-fit test, and the number of covariates was kept limited in view of the 45 OGTT-tested participants. As some women in the OGTT-untested group may not yet have completed the routine GDM screening window, and given that this window is generally 24–28 weeks of gestation, the primary analyses were repeated as a sensitivity analysis restricted to women who had completed this window (≥28 gestational weeks), while the full sample was retained as the primary analysis.

Multiple linear regression analysis (entry method) was applied to identify variables associated with the total e-health literacy score. Variables were selected for the model based on their association with eHEALS scores in univariate analyses (*p* < 0.20). In addition, OGTT status was included a priori because it represented the primary independent variable of interest. Categorical variables were dummy-coded. The model assumptions were met in terms of the normality of the residuals, the independence of the error terms (Durbin-Watson = 1.983), and the absence of multicollinearity (variance inflation factor < 2). The sample sizes obtained (*n* = 45 and *n* = 122) were sufficient to detect a medium-sized effect (Cohen’s d = 0.50) with 81.3% power at the α = 0.05 level. A two-tailed *p*-value < 0.05 was considered significant. Employment status data were missing for one participant. Analyses involving this variable were performed using available data (*n* = 166), and no imputation was performed.

## 3. Results

### 3.1. Demographic Characteristics of Participants

A total of 167 pregnant women were included in the study. At study enrollment, 45 (26.9%) were classified as OGTT-tested and 122 (73.1%) as OGTT-untested. The mean age of the sample was 28.62 ± 5.30 years (range 18–42), and the median gestational week was 29.0 (range 23–42). Of the participants, 165 (98.8%) were married, and all reported that they were not currently taking psychiatric medications and were not using alcohol or drugs.

The distribution of sociodemographic and obstetric characteristics by group is presented in Table 1. No significant differences were found between the two groups in terms of age, age group, educational level, place of residence, perceived economic status, employment status, gravida, gravida group, gestational age, trimester, comorbid organic disease, history of psychiatric diagnosis, and smoking status (all *p* > 0.05). In contrast, the proportion of women who had undergone an OGTT in a previous pregnancy was higher in the OGTT-tested group (20/45) than in the OGTT-untested group (16/122) (*p* < 0.001) (Table 1).

### 3.2. Internet Use and Levels of E-Health Literacy

Of the participants, 129 (77.2%) reported using the internet to obtain health information, and 82 (49.1%) stated that they trusted the health information they found online. For the two items on the scale that were not included in the scoring, the median response was 4.0 (1–5) for both items.

The mean total score from the e-Health Literacy Scale was 24.71 ± 8.58 (8–40) for the entire sample. In the present sample, the internal consistency of the scale was excellent (Cronbach’s alpha = 0.94). The total score was 25.04 ± 8.20 in the OGTT-tested group and 24.58 ± 8.75 in the OGTT-untested group; the difference between the two groups was not statistically significant (Mann–Whitney U test, *p* = 0.788). The Hodges–Lehmann median difference between the groups was 0.00 points (95% CI: −3.00 to 4.00), and the effect size was negligible (r = 0.027; 95% CI: −0.163 to 0.226). No significant differences were found between the groups regarding the use of the Internet to obtain health information (*p* = 0.600), trust in health information found online (*p* = 0.109), or responses to the two items not included in the scoring (*p* = 0.161 and *p* = 0.300, respectively) (Table 2).

### 3.3. Distribution of E-Health Literacy Scores by Participant Characteristics

A comparison of the total e-Health literacy scores according to participant characteristics is presented in Table 3. The scores differed by age group (*p* = 0.019), educational level (*p* < 0.001), and trimester (*p* = 0.014). The difference observed across age groups stemmed from the contrast between pregnant women aged 35 and older and those under 25; the highest score was obtained in the 35 and older group (27.86 ± 8.24), while the lowest score was observed in the under-25 group (22.20 ± 7.91) (ε^2^ = 0.048). The difference observed in educational level stemmed from the fact that the score of pregnant women with a university degree (29.10 ± 8.29) was higher than that of pregnant women with a high school education (22.47 ± 8.59) and those with an elementary school education or lower (24.14 ± 7.67) (ε^2^ = 0.111). In the comparison by trimester, the scores of pregnant women in the second trimester (26.83 ± 9.55) were found to be higher than those of pregnant women in the third trimester (23.69 ± 7.92) (r = 0.236) (Table 3).

No significant differences in e-Health literacy scores were found based on the variables of whether an OGTT was performed, place of residence, perceived economic status, employment status, gravidity group, having undergone an OGTT in a previous pregnancy, using the internet to obtain health information, and trust in online health information (*p* = 0.788; *p* = 0.627; *p* = 0.427; *p* = 0.933; *p* = 0.061; *p* = 0.542; *p* = 0.125, and *p* = 0.056) (Table 3).

### 3.4. Variables Associated with the E-Health Literacy Score

Total e-health literacy score; age (rho = 0.240; *p* = 0.002), the first item of the scale (which was not included in the scoring; rho = 0.320; *p* < 0.001), and the second item (rho = 0.318; *p* < 0.001), and was negatively correlated with gestational week (rho = −0.225; *p* = 0.004). No significant association was found with gravida (rho = 0.069; *p* = 0.378).

The model established in the multiple linear regression analysis was statistically significant (F = 3.835; *p* < 0.001) and explained 12.0% of the variation in e-health literacy scores (R^2^ = 0.163; adjusted R^2^ = 0.120). In the model, having a college degree (B = 4.079; 95% CI: 0.244–7.915; *p* = 0.037) and trusting health information on the internet (B = 3.466; 95% CI: 0.421–6.511; *p* = 0.026) were found to be independently associated with the score. The variables of OGTT status, age, gestational week, multigravity, and using the internet to obtain health information did not make a significant contribution to the model (all *p* > 0.05) (Table 4).

#### Factors Associated with OGTT Status at Study Enrollment

A binary logistic regression analysis was performed with OGTT status at study enrollment as the dependent variable, entering the eHEALS score together with age, education level, gestational week, previous-pregnancy OGTT, and trust in online health information. The model was statistically significant (likelihood-ratio χ^2^ = 22.77; *p* = 0.002; McFadden pseudo-R^2^ = 0.117) and showed adequate fit (Hosmer–Lemeshow *p* = 0.989). Having undergone an OGTT in a previous pregnancy was independently associated with being OGTT-tested in the current pregnancy (OR = 5.43; 95% CI: 2.40–12.30; *p* < 0.001), and trust in online health information was inversely associated with OGTT-tested status (OR = 0.44; 95% CI: 0.20–0.97; *p* = 0.042). The eHEALS score was not associated with OGTT status (OR = 1.02; 95% CI: 0.98–1.08; *p* = 0.321) (Table 5). In a sensitivity analysis restricted to women who had completed the routine screening window (≥28 gestational weeks; *n* = 113), the findings were essentially unchanged: the eHEALS comparison between groups remained non-significant (*p* = 0.807), and in the logistic regression, previous-pregnancy OGTT (OR = 12.14; 95% CI: 4.01–36.76; *p* < 0.001) and trust in online information (OR = 0.24; 95% CI: 0.08–0.72; *p* = 0.011) remained significant, while the eHEALS score remained non-significant (OR = 1.05; *p* = 0.183).

### 3.5. OGTT Findings Among Women in the OGTT-Tested Group

Among 45 pregnant women who underwent a 75 g OGTT during their current pregnancy, the mean fasting plasma glucose level was 84.24 ± 8.86 mg/dL, the one-hour value was 130.49 ± 32.86 mg/dL, and the two-hour value was 111.58 ± 19.64 mg/dL (*n* = 43). According to IADPSG criteria, 14 of these pregnant women (31.1%) were diagnosed with GDM. In an exploratory subgroup analysis, e-health literacy scores were higher among women diagnosed with GDM than among those without GDM (*p* = 0.042) (Table 3). Given the small subgroup sizes, this finding should be interpreted cautiously. No significant correlation was found between fasting, one-hour, and two-hour glucose levels from the OGTT and the e-Health literacy score (rho = 0.101; *p* = 0.509, rho = 0.020; *p* = 0.897, and rho = −0.168; *p* = 0.280, respectively).

**Figure 1:**
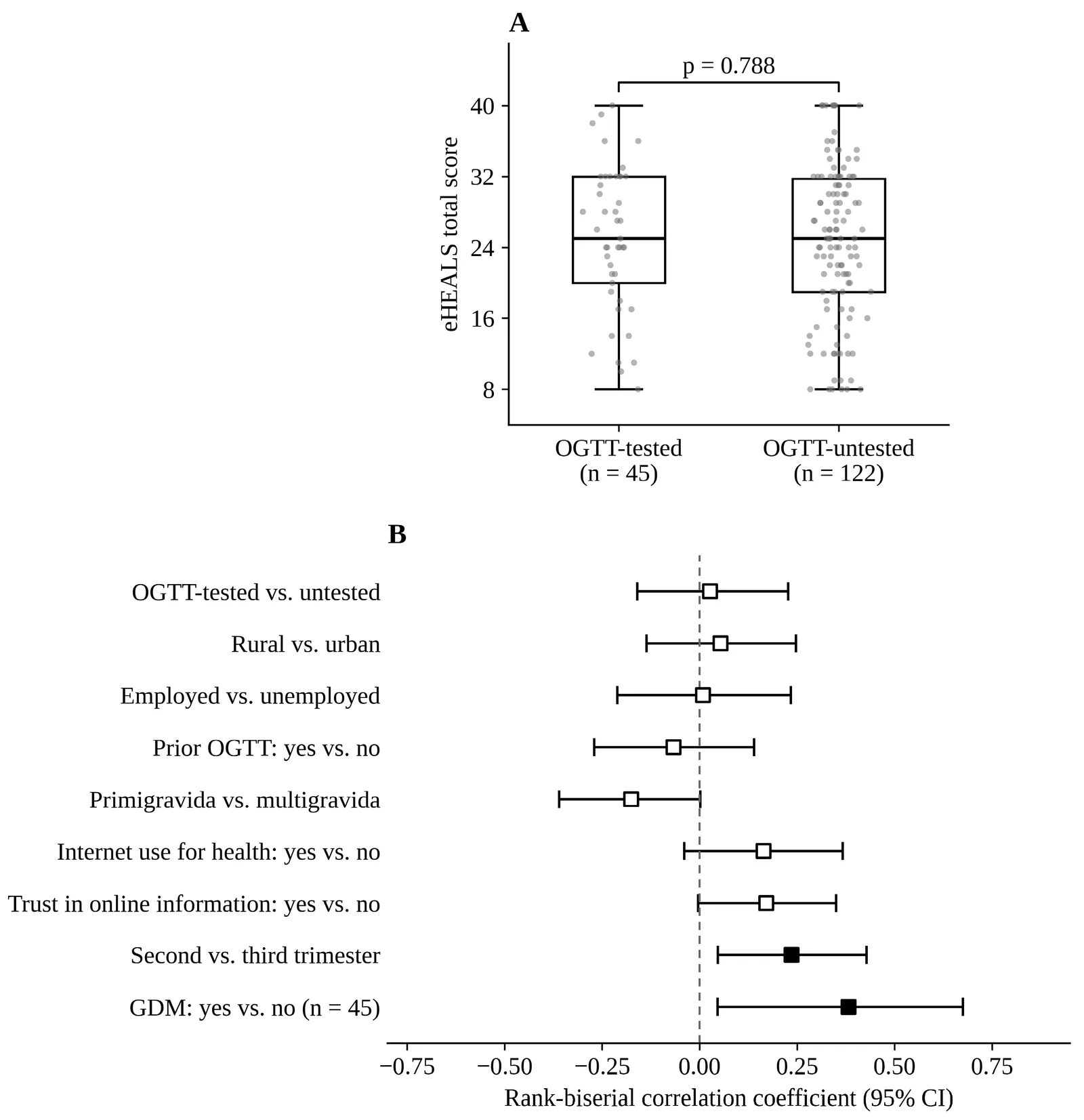
(**A**) Distribution of total e-Health Literacy Scale scores according to OGTT status at study enrollment; boxes represent the interquartile range, horizontal lines within the boxes indicate the median, and dots represent individual observations. (**B**) Rank-biserial correlation coefficients and 95% confidence intervals for pairwise comparisons of e-Health literacy scores; filled squares indicate comparisons in which the confidence interval does not include zero. The GDM comparison was performed only among pregnant women who underwent OGTT during the current pregnancy (*n* = 45).

The distribution of the total e-Health literacy score by group and the effect sizes for the pairwise comparisons examined in the study are shown in Figure 1.

## 4. Discussion

In our study, we evaluated the association between e-health literacy and OGTT status at the time of study enrollment and found that the total eHEALS scores were similar between the groups that underwent OGTT and those that did not. No significant difference was found between the groups, and the observed effect size was determined to be negligible.

The lack of a significant association between e-health literacy and OGTT status at study enrollment can be explained by various factors. OGTT status at study enrollment may be associated with factors beyond e-health literacy, including prior experiences and other individual and health care system-related factors. Additionally, the heterogeneous nature of the group that did not undergo the OGTT and the fact that eHEALS was administered to some participants after the OGTT or a GDM diagnosis may have made it difficult to interpret the relationship between the two.

Uyanıklar et al. reported that general health literacy scores were higher among the 364 pregnant women who agreed to undergo GDM screening and emphasized that low health literacy was associated with an increased likelihood of refusing screening [10]. Erdem et al. noted that the choice of an OGTT is not associated with health literacy or anxiety levels during the perinatal period, but that healthcare professionals play an important role in pregnant women’s decision to participate [18]. However, these studies assess general health literacy. e-Health literacy, on the other hand, reflects an individual’s perceived competence in accessing and using online information [26]. In a study that directly examined the relationship between e-health literacy and health behaviors during pregnancy, the average eHEALS score was found to be 29.37 ± 6.20, and it was reported that e-health literacy, the use of health information from the internet, and gestational age are predictors of healthy lifestyle behaviors [27]. In a study conducted in Denmark involving 405 pregnant women, e-health literacy was generally lower among participants who were immigrants or of immigrant origin; the difference was found to be particularly pronounced in the ability to interact with digital services [28]. In China, a strong positive association was found between eHEALS and self-efficacy in third-trimester pregnant women, and it was noted that the relationship between self-efficacy, e-health literacy, and preparation for childbirth may be significant [29]. A review article examined the relationship between internet use during pregnancy and health literacy. This article found that higher health literacy was associated with the frequency of online information searches, reliance on official sources, and discussing the information obtained with a healthcare professional, while low literacy was reported to be associated with difficulties in understanding the information [30]. Upon reviewing the literature, we found only a limited number of studies that assessed e-health literacy for GDM screening tests, which can simultaneously affect the health of two individuals. From this perspective, we believe our study makes significant contributions.

Although a significant proportion of pregnant women reported using the internet for health information, it is noteworthy that only half of them stated that they trusted the information they found online. The fact that trust in online health information is independently associated with e-health literacy scores in the multivariate model supports this distinction. While internet use represents access to information, the variable of trust in the information obtained may be more closely related to an individual’s perceived self-efficacy. However, this finding should not be interpreted causally. A study conducted among women diagnosed with GDM also reported that obtaining information from healthcare professionals and mobile apps was associated with higher e-health literacy; however, it was noted that women with GDM faced various barriers in accessing online information and using these apps [31]. Therefore, in clinical practice, it is important not to overlook which sources pregnant women use, how they verify information, and how they handle content that conflicts with healthcare provider recommendations.

Our findings suggest that e-health literacy is associated with certain sociodemographic and pregnancy-related characteristics. While the scores of pregnant women aged 35 and older were statistically significantly higher than those of the group under 25, we found that age was not an independent predictor. This suggests that the statistically significant age difference may be explained by education, digital experience, or other health-related variables. Guo et al. reported that age is one of the independent predictors of e-health literacy and that younger women tend to have higher scores. This difference may be related to differences in the samples and in variables related to access to digital services. Similarly, in a study conducted by Guo et al. among pregnant women diagnosed with GDM, educational level was identified as one of the independent predictors of e-health literacy, and it was reported that a higher educational level was associated with higher e-health literacy scores [20]. Unlike our study, this study included only pregnant women diagnosed with GDM. The significant difference in the initial analysis supports the idea that educational level may be a stronger indicator of differences in the ability to access and evaluate online health information. Although the regression model was statistically significant, it explained only a limited proportion of the variance in e-health literacy scores; this suggests that other unmeasured factors may also contribute to e-health literacy.

The finding that e-health literacy scores were higher in the second trimester than in the third trimester suggests that the way women seek and use health information may change as pregnancy progresses. Indeed, Szwajcer et al. reported that the sources of nutrition information women consulted and the content they sought differed depending on the stage of pregnancy [32]. However, this study did not compare e-health literacy scores across trimesters. Although this appears to corroborate our findings, due to the cross-sectional design of our study, it cannot be concluded that this is a direct effect of gestational week. Since the types of online information pregnant women seek may vary during the third trimester, longitudinal studies that follow the same women throughout their pregnancies are necessary to confirm trimester differences.

Previous-pregnancy OGTT was strongly associated with OGTT-tested status in the current pregnancy. However, our study did not assess the nature of the previous testing experience, whether the test result was normal or abnormal, or the pregnant women’s perceptions of that experience. Previous OGTT experience may be associated with greater familiarity with the test and with OGTT status in the current pregnancy.

In a subgroup analysis conducted for exploratory purposes, although e-health literacy scores were found to be higher among pregnant women diagnosed with GDM compared to those not diagnosed with GDM, this finding should be interpreted with caution due to the small size of the subgroups and needs to be confirmed by studies with larger sample sizes. However, the cross-sectional design of our study does not allow us to conclude that high e-health literacy is associated with the development of GDM. Women diagnosed with GDM may have begun seeking more information about their condition after receiving the diagnosis.

### Strengths and Limitations

A strength of this study is that it assessed the association between e-health literacy and OGTT status at study enrollment, taking into account relevant sociodemographic and obstetric variables. Limitations of this study include its cross-sectional design and the small number of participants in the group that underwent an OGTT, particularly the subgroup diagnosed with GDM. It should be noted that the group that did not undergo an OGTT may be heterogeneous in terms of clinical and behavioral characteristics, and that the reasons for not performing the OGTT were not systematically recorded. Therefore, the heterogeneity of the OGTT-untested group may have limited the comparability of the groups and introduced potential selection bias. The timing of the eHEALS assessment is another limitation of our study. In the group that underwent the OGTT, the eHEALS assessment was conducted after the OGTT; in pregnant women with GDM, it was conducted after the diagnosis was made. Therefore, undergoing the OGTT or receiving a diagnosis of GDM may have influenced subsequent health information-seeking behavior and perceived e-health literacy. Some women in the group that did not undergo an OGTT may not yet have reached the recommended stage of pregnancy for GDM screening. In addition, factors that could influence the decision to administer an OGTT such as a physician’s recommendation, a family history of diabetes, pregnancy risk status, and adverse pregnancy outcomes were not evaluated in our study. Furthermore, eHEALS is not an objective assessment of digital health literacy but rather a self-report measure of perceived e-health literacy. Therefore, high eHEALS scores may not always reflect a person’s actual ability to identify reliable online information, evaluate digital resources, or translate that information into health-related behaviors. As eHEALS was assessed after OGTT among OGTT-tested women, the temporal direction of the association cannot be established, and reverse causality cannot be excluded. Although the Turkish version of the eHEALS used in this study was found to be valid and reliable by Uskun et al. [24], the Turkish validity study was conducted among adults aged 45 and older; the participants in our study, however, were between the ages of 18 and 42. Therefore, the measurement validity of this version of the scale among younger pregnant women has not been specifically established, and this should be taken into account when interpreting the results.

## 5. Conclusions

In conclusion, in this sample, the level of e-health literacy did not differ significantly according to OGTT status at study enrollment. In contrast, having a college degree and trusting online health information were found to be independently associated with higher levels of e-health literacy. These findings indicate that e-health literacy was not significantly associated with OGTT status at study enrollment in this sample. Furthermore, given the study’s cross-sectional design and the limited sample size of the group that underwent OGTT, the findings should be interpreted with caution. Multicenter, larger-scale, and prospective studies will contribute to a more comprehensive understanding of e-health literacy in the context of GDM, attitudes and beliefs regarding the OGTT, the quality of digital information sources, and factors associated with OGTT status.

## Figures and Tables

**Table 1:** Distribution of sociodemographic and obstetric characteristics by group.

Variable		OGTT-Tested (*n* = 45)	OGTT-Untested (*n* = 122)	Total (*n* = 167)	Effect Size (95% CI)	*p*
Age (years), Mean ± SD		28.93 ± 5.35	28.51 ± 5.29	28.62 ± 5.30	d = 0.080 (−0.269; 0.412)	0.647
Age group, *n* (%)	<25 age	11 (24.4)	29 (23.8)	40 (24.0)	V = 0.058	0.756
	25–34 age	27 (60.0)	79 (64.8)	106 (63.5)		
	≥35 age	7 (15.6)	14 (11.5)	21 (12.6)		
Education level, *n* (%)	Primary education or below	13 (28.9)	44 (36.1)	57 (34.1)	V = 0.068	0.683
	High school	20 (44.4)	48 (39.3)	68 (40.7)		
	University	12 (26.7)	30 (24.6)	42 (25.1)		
Place of residence, *n* (%)	Rural	6 (13.3)	28 (23.0)	34 (20.4)	V = 0.106	0.249
	Urban	39 (86.7)	94 (77.0)	133 (79.6)		
Economic status, *n* (%)	Low	3 (6.7)	21 (17.2)	24 (14.4)	V = 0.136	0.212
	Middle	37 (82.2)	91 (74.6)	128 (76.6)		
	High	5 (11.1)	10 (8.2)	15 (9.0)		
Employment status, *n* (%) *	Employed	12 (26.7)	29 (24.0)	41 (24.7)	V = 0.028	0.876
	Unemployed	33 (73.3)	92 (76.0)	125 (75.3)		
Gravida, median (min–max)		2.0 (1–5)	2.0 (1–7)	2.0 (1–7)	r = 0.008 (−0.179; 0.197)	0.936
Gravida group, *n* (%)	Primigravida	16 (35.6)	44 (36.1)	60 (35.9)	V = 0.005	1.000
	Multigravida	29 (64.4)	78 (63.9)	107 (64.1)		
Gestational age, median (min–max)		29.0 (24–38)	29.0 (23–42)	29.0 (23–42)	r = 0.032 (−0.157; 0.222)	0.753
Trimester, *n* (%)	Second trimester (<28 weeks)	14 (31.1)	40 (32.8)	54 (32.3)	V = 0.016	0.985
	Third trimester (≥28 weeks)	31 (68.9)	82 (67.2)	113 (67.7)		
OGTT performed in a previous pregnancy, *n* (%)	Yes	20 (44.4)	16 (13.1)	36 (21.6)	V = 0.338	**<0.001**
	No	25 (55.6)	106 (86.9)	131 (78.4)		
Comorbid organic disease (present), *n* (%)		3 (6.7)	9 (7.4)	12 (7.2)	V = 0.012	1.000
History of psychiatric diagnosis (present), *n* (%)		1 (2.2)	4 (3.3)	5 (3.0)	V = 0.028	1.000
Smoking (yes), *n* (%)		2 (4.4)	11 (9.0)	13 (7.8)	V = 0.076	0.517

Mean: mean; SD: standard deviation; CI: confidence interval; OGTT: oral glucose tolerance test. Effect sizes: d = Cohen’s d; r = rank-biserial correlation coefficient; V = Cramér’s V. Percentages are column percentages. * Employment status data were missing for one participant; therefore, *n* = 166 for this variable. Significant *p*-values are shown in bold.

**Table 2:** Comparison of Internet usage characteristics and e-Health literacy scores by group.

Variable		OGTT-Tested (*n* = 45)	OGTT-Untested (*n* = 122)	Total (*n* = 167)	Effect Size (95% CI)	*p*
Use of the Internet to obtain health information (yes), *n* (%)		33 (73.3)	96 (78.7)	129 (77.2)	V = 0.057	0.600
Trust in health information on the Internet (yes), *n* (%)		17 (37.8)	65 (53.3)	82 (49.1)	V = 0.138	0.109
Perceived usefulness of Internet assistance **, median (min–max)		3.0 (1–5)	4.0 (1–5)	4.0 (1–5)	r = −0.134 (−0.310; 0.031)	0.161
Importance of access to health resources **, median (min–max)		4.0 (1–5)	4.0 (1–5)	4.0 (1–5)	r = −0.101 (−0.290; 0.090)	0.300
eHEALS total score	Mean ± SD	25.04 ± 8.20	24.58 ± 8.75	24.71 ± 8.58	r = 0.027 (−0.150; 0.221)	0.788
	Median (min–max)	25.0 (8–40)	25.0 (8–40)	25.0 (8–40)		

Mean: mean; SD: standard deviation; CI: confidence interval; eHEALS: eHealth Literacy Scale; OGTT: oral glucose tolerance test. Effect sizes: r = rank-biserial correlation coefficient; V = Cramér’s V. Percentages are column percentages. ** Two items of the scale that are not included in the total score (1: not useful at all/not important at all; 5: very useful/very important).

**Table 3:** Comparison of the total e-Health literacy score by participant characteristics.

Variable		*n*	Mean ± SD	Median (Min–Max)	Effect Size (95% CI)	*p*
OGTT status	Tested	45	25.04 ± 8.20	25.0 (8–40)	r = 0.027 (−0.160; 0.227)	0.788
	Not Tested	122	24.58 ± 8.75	25.0 (8–40)		
Age group	<25 age	40	22.20 ± 7.91 ^a^	22.5 (8–40)	ε^2^ = 0.048 (0.010; 0.131)	**0.019**
	25–34 age	106	25.03 ± 8.71 ^ab^	26.0 (8–40)		
	≥35 age	21	27.86 ± 8.24 ^b^	29.0 (9–40)		
Education level	Primary education or below	57	24.14 ± 7.67 ^a^	24.0 (8–40)	ε^2^ = 0.111 (0.043; 0.231)	**<0.001**
	High school	68	22.47 ± 8.59 ^a^	23.0 (8–40)		
	University	42	29.10 ± 8.29 ^b^	31.5 (8–40)		
Place of residence	Rural	34	25.71 ± 6.53	25.0 (12–40)	r = 0.054 (−0.136; 0.247)	0.627
	Urban	133	24.45 ± 9.03	25.0 (8–40)		
Economic status	Low	24	22.88 ± 8.44	22.0 (8–40)	ε^2^ = 0.010 (0.001; 0.076)	0.427
	Middle	128	25.15 ± 8.37	26.0 (8–40)		
	High	15	23.87 ± 10.58	25.0 (8–40)		
Employment status *	Employed	41	24.29 ± 9.87	27.0 (8–40)	r = 0.009 (−0.211; 0.234)	0.933
	Unemployed	125	24.78 ± 8.16	25.0 (8–40)		
Gravida group	Primigravida	60	23.18 ± 8.69	24.0 (8–40)	r = −0.175 (−0.360; 0.002)	0.061
	Multigravida	107	25.56 ± 8.44	26.0 (8–40)		
Trimester	Second trimester (<28 weeks)	54	26.83 ± 9.55	29.0 (8–40)	r = 0.236 (0.047; 0.428)	**0.014**
	Third trimester (≥28 weeks)	113	23.69 ± 7.92	24.0 (8–40)		
OGTT performed in a previous pregnancy	Yes	36	23.67 ± 8.68	26.0 (8–40)	r = −0.067 (−0.270; 0.140)	0.542
	No	131	24.99 ± 8.56	25.0 (8–40)		
Use of the Internet for health information	Yes	129	25.22 ± 8.63	26.0 (8–40)	r = 0.164 (−0.039; 0.367)	0.125
	No	38	22.95 ± 8.29	23.0 (8–40)		
Trust in health information on the Internet	Yes	82	25.98 ± 8.33	27.0 (8–40)	r = 0.171 (−0.004; 0.350)	0.056
	No	85	23.48 ± 8.69	24.0 (8–40)		
GDM diagnosis Present Absent	Yes	14	28.93 ± 6.26	28.0 (19–39)	r = 0.382 (0.046; 0.675)	**0.042**
	No	31	23.29 ± 8.45	24.0 (8–40)		

Mean: mean; SD: standard deviation; CI: confidence interval; OGTT: oral glucose tolerance test. Effect sizes: r = rank-biserial correlation coefficient; ε^2^ = epsilon-squared. ^a,b^ Groups with different superscript letters differ significantly in pairwise comparisons (Bonferroni-corrected Mann–Whitney U test). * Employment status data were missing for one participant; therefore, *n* = 166 for this variable. Significant *p*-values are shown in bold.

**Table 4:** Variables predicting the total e-Health literacy score: multiple linear regression analysis.

Independent Variable	B	95% CI	β	t	*p*
Constant	24.626	11.921–37.332	–	3.828	**<0.001**
Age (years)	0.142	−0.127–0.410	0.087	1.042	0.299
Education: High school †	−1.595	−4.669–1.479	−0.092	−1.025	0.307
Education: University †	4.079	0.244–7.915	0.207	2.100	**0.037**
Gestational age	−0.236	−0.558–0.085	−0.116	−1.453	0.148
Multigravida ‡	1.989	−0.869–4.847	0.112	1.375	0.171
Trust in health information on the Internet (yes)	3.466	0.421–6.511	0.203	2.248	**0.026**
Use of the Internet for health information (yes)	−0.735	−4.505–3.034	−0.036	−0.385	0.700
Underwent OGTT §	0.914	−1.905–3.732	0.047	0.640	0.523

B: Unstandardized regression coefficient; CI: confidence interval; β: standardized regression coefficient; OGTT: oral glucose tolerance test. Model: F = 3.835; *p* < 0.001; R^2^ = 0.163; adjusted R^2^ = 0.120; Durbin–Watson = 1.983; variance inflation factor <2 for all variables. † Reference category: primary education or below. ‡ Reference category: primigravida. § Reference category: did not undergo OGTT. Significant *p*-values are shown in bold.

**Table 5:** Binary logistic regression analysis of factors associated with OGTT status at study enrollment.

Independent Variable	B (SE)	OR (95% CI)	Wald	*p*
Constant	−3.265 (2.018)	0.04 (0.00–1.99)	2.62	0.106
e-Health literacy score	0.024 (0.025)	1.02 (0.98–1.08)	0.99	0.321
Age (years)	0.001 (0.039)	1.00 (0.93–1.08)	0.00	0.977
Education: High school †	0.484 (0.456)	1.62 (0.66–3.96)	1.12	0.289
Education: University †	0.370 (0.544)	1.45 (0.50–4.21)	0.46	0.497
Gestational age	0.042 (0.049)	1.04 (0.95–1.15)	0.74	0.391
OGTT in a previous pregnancy (yes) ‡	1.691 (0.417)	5.43 (2.40–12.30)	16.43	**<0.001**
Trust in health information on the Internet (yes) §	−0.831 (0.408)	0.44 (0.20–0.97)	4.14	**0.042**

B: Regression coefficient; SE: standard error; OR: odds ratio; CI: confidence interval; OGTT: oral glucose tolerance test. Dependent variable: OGTT status at study enrollment (1 = OGTT-tested; 0 = OGTT-untested). † Reference category: primary education or below. ‡ Reference category: no. § Reference category: no. Model: likelihood-ratio χ^2^ = 22.77; *p* = 0.002; McFadden pseudo-R^2^ = 0.117; Hosmer–Lemeshow *p* = 0.989; *n* = 167. Significant *p*-values are shown in bold.

## Data Availability

The data presented in this study are available from the corresponding author upon reasonable request.
